# Temporal Trends in Geographical Variation in Breast Cancer Mortality in China, 1973–2005: An Analysis of Nationwide Surveys on Cause of Death

**DOI:** 10.3390/ijerph13100963

**Published:** 2016-09-28

**Authors:** Changfa Xia, Clare Kahn, Jinfeng Wang, Yilan Liao, Wanqing Chen, Xue Qin Yu

**Affiliations:** 1 National Office for Cancer Prevention and Control, National Cancer Center/Cancer Hospital, Chinese Academy of Medical Sciences and Peking Union Medical College, Beijing 100021, China; xiacfa@163.com; 2 Cancer Council New South Wales, Sydney NSW 2011, Australia; clarek@nswcc.org.au; 3 State Key Laboratory of Resources and Environmental Information System, Institute of Geographic Sciences and Natural Resources Research, Chinese Academy of Sciences, Beijing 100101, China; wangjf@lreis.ac.cn (J.W.); liaoyl@lreis.ac.cn (Y.L.); 4 Sydney School of Public Health, University of Sydney, Sydney NSW 2006, Australia

**Keywords:** breast cancer, mortality, temporal, geographical, China

## Abstract

To describe geographical variation in breast cancer mortality over time, we analysed breast cancer mortality data from three retrospective national surveys on causes of death in recent decades in China. We first calculated the age-standardized mortality rate (ASMR) for each of the 31 provinces in mainland China stratified by survey period (1973–1975, 1990–1992 and 2004–2005). To test whether the geographical variation in breast cancer mortality changed over time, we then estimated the rate ratio (RR) for the aggregated data for seven regions and three economic zones using generalized linear models. Finally, we examined the correlation between mortality rate and several macro-economic measures at the provincial level. We found that the overall ASMR increased from 2.98 per 100,000 in 1973–1975 to 3.08 per 100,000 in 1990–1992, and to 3.85 per 100,000 in 2004–2005. Geographical variation in breast cancer mortality also increased significantly over time at the regional level (*p* = 0.002) but not at the economic zone (*p* = 0.089) level, with RR being generally lower for Western China (Northwest and Southwest) and higher in Northeast China over the three survey periods. These temporal and spatial trends in breast cancer mortality were found to be correlated with per capita gross domestic product, number of hospitals and health centres’ beds per 10,000 population and number of practicing doctors per 10,000 population, and average number of live births for women aged 15–64. It may be necessary to target public health policies in China to address the widening geographic variation in breast cancer mortality, and to take steps to ensure that the ease of access and the quality of cancer care across the country is improved for all residents.

## 1. Introduction

Breast cancer is the most commonly diagnosed cancer among women worldwide, and the leading cause of cancer death among women in less developed regions [1]. In China, breast cancer is now the most frequently diagnosed cancer and the sixth leading cause of cancer-related death in women, with 273,000 Chinese women being diagnosed with breast cancer in 2012 and 62,000 dying of the disease [2]. While the incidence rate of breast cancer in China is still lower than is reported in developed countries, since the 1990s, breast cancer incidence in China has increased at a rate more than double the increases seen in global rates [3].

Since the late 1970s, when the initiation of economic reforms in China began to lead to a shift from a predominately rural lifestyle to a more urban lifestyle, breast cancer incidence and mortality rates in China have been gradually increasing [4]. These economic developments, along with accelerating urbanization and the adoption of a Westernized lifestyle, are believed to have contributed greatly to the overall increase in breast cancer incidence over time in China [4,5,6]. It also seems likely that these economic developments have significantly increased inequalities in breast cancer mortality within China due to uneven development across geographical regions [7,8,9]. In a recent study that compared the results of two death surveys in China, Zhou and colleagues found that the areas of highest breast cancer mortality shifted from being in the large cities (Beijing and Shanghai) in the 1970s to being in more rural areas of the provinces in Northeast China by the period 2004–2005 [10]. There are, however, some limitations to most previous studies of geographical variation in breast cancer mortality, as they tended to either use data from the 1990s to 2010s [7,8], and thus provide no information on breast cancer in the 1980s and later 1970s when the Chinese economic reforms began, or they use data from only two time periods [9].

To begin to address these concerns this study aimed to provide a detailed analysis of the geographical variation and temporal changes in female breast cancer mortality in China from 1973 to 2005. Data from three nationally representative death surveys were used to detect the temporal trends in geographical variation in breast cancer mortality and to explore the relationship between the distribution of breast cancer mortality and selected socioeconomic factors such as per capita gross domestic product (GDP), number of hospitals and health centers’ beds (NHHCB) per 10,000 population, number of practicing doctors (NPD) per 10,000 population and average number of live births for women aged 15–64.

## 2. Materials and Methods 

### 2.1. Data Sources

Breast cancer mortality data were extracted from three national retrospective surveys on cause of death, conducted by the Chinese Ministry of Health in 1973–1975, 1990–1992 and 2004–2005. The data included the number and age of people dying from breast cancer, and the population of each geographical area. The three surveys of cause of death in China have been described in detail in previous publications [11,12,13]. Briefly, the First National Survey of Death Causes for the period 1973–1975 covered nearly all counties/cities/districts of mainland China (excluding Taiwan, Hong Kong, Macau, and 35 sparsely populated counties), with a total population of 850 million [11]. Using a retrospective method, this survey completed an ad hoc investigation of causes of all deaths in nearly all households in China during 1973–1975. The Second National Retrospective Sampling Survey of Death Causes for the period 1990–1992 included 263 sample points so that a total of 335 million person-years were investigated, accounting for 9.8% of the total national population [12]. The Third National Retrospective Sampling Survey of Death Causes for the period 2004–2005 covered 158 counties/cities/districts, accounting for 143 million person-years between 2004 and 2005 [13]. These two sampling surveys collected information from a nationally representative sample and recorded deaths which occurred in the study years. As this is published tabulated data, no ethics approval was required for this study.

Macro data on several socioeconomic development levels were used to examine the correlation between breast cancer mortality and economic development. The data of per capita GDP were derived from the China Compendium of Statistics 1949–2008 [14]. The NHHCB per 10,000 population, and NPD per 10,000 population for 1975 and 1992 were taken from the National Bureau of Statistics of the People’s Republic of China [15], and, for 2005, they were taken from the 2006 China Health Statistical Yearbook [16]. The data of average number of live births for women aged 15–64 were extracted from the tabulation of the three national population censuses collected from 1982 to 2000: the Third (1982), the Fourth (1990), and the Fifth National Population Census (2000).

### 2.2. Geographical Unit Used in Analyses

The basic geographical unit used for analyses in this study is province, or autonomous region or municipality of Beijing, Tianjin, Shanghai and Chongqing.

**Figure 1:**
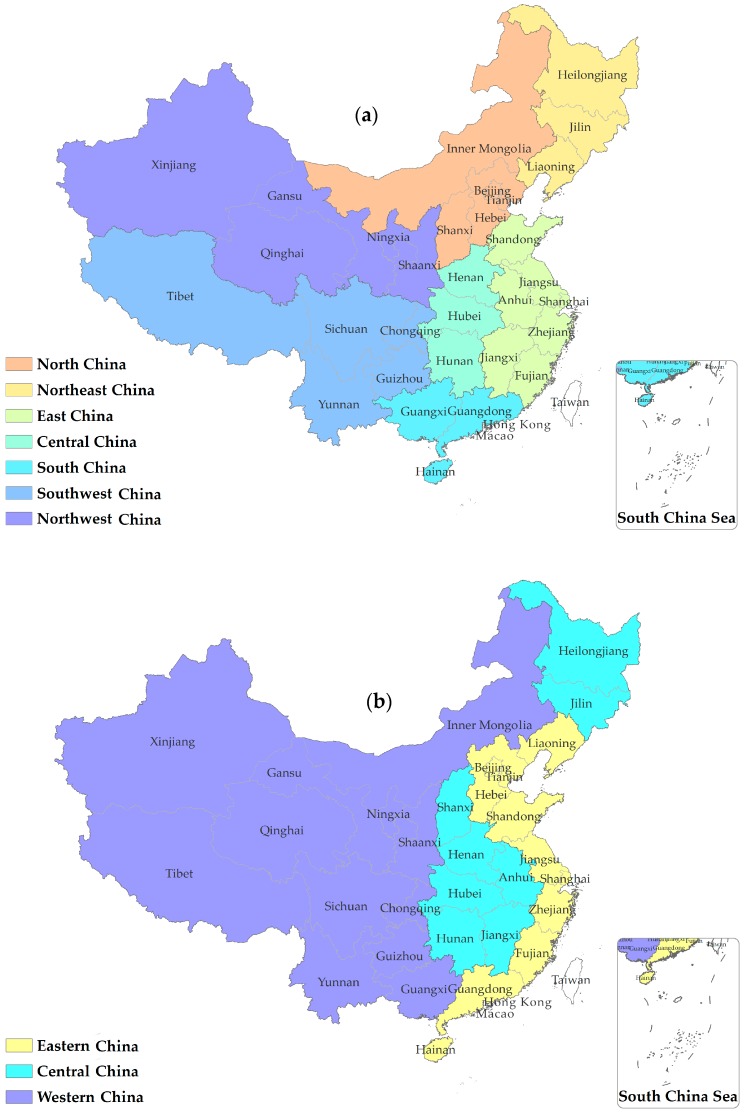
Schematic diagram of the division of China into the geographical units used for analyses showing (**a**) the seven geographical regions; and (**b**) three economic zones. Map source: Sino Maps Press.

We divided mainland China into seven geographical regions (Figure 1a) and three economic zones (Figure 1b). The list of provinces included in these regions or zones can be found in online Appendix A.

### 2.3. Data Analysis

We first calculated age-standardized morality rates (ASMR), using the direct standardization method (to the 1982 female Chinese standard population), for each province and each of the three cause of death surveys. These province-based data were then aggregated into seven geographical regions and three economic zones, respectively.

We used generalized linear models with a negative binomial error structure (to overcome over-dispersion [17]) to calculate the rate ratio (RR) of death from breast cancer for the aggregated data for region and zone. We calculated the RR of death for each geographical unit stratified by cause of death survey (1972–1975, 1990–1992 and 2004–2005). In this model, the main-effect variables were geographical unit (seven regions or three zones), age group at death (<25 years, 25–29 years,…, 75–79 years, ≥80 years), and the natural logarithm of the population size as the offset variable. The RR derived from this model is the ratio of death from breast cancer in a given geographical unit to that of the reference (the national average of each survey). Ninety-five percent confidence intervals (CIs) for the geographical units were calculated using the estimated coefficients and standard errors from the negative binomial regression model. We then added an interaction term for geographical unit and time period to the model, to allow the effect of geographical unit to change between periods, and then used a likelihood ratio test between the nested models to determine if this interaction was significant [18,19].

Correlation of the provincial mortality rate of breast cancer and provincial macro-economic measures was calculated separately for each survey period. In order to avoid any confounding caused by age, we used crude rate rather than age-adjusted rate.

All significance tests with *p*-value < 0.05 were taken to indicate statistical significance. Statistical analyses were performed using SAS software (version 9.3, Institute Inc., Cary, NC, USA). All maps were created using ESRI (Environmental Systems Research Institute, Inc., Redlands, CA, USA) ArcGIS 10.2 software.

## 3. Results

### 3.1. Age-Standardized Mortality Rate

Detailed data including the numbers of breast cancer deaths and corresponding populations by province and survey period can be found in online Appendix B Table B1. The economic measure of per capita GPD by province is also included.

From the mid-1970s to the mid-2000s, there was an increase in female breast cancer mortality in China. Average crude mortality rates (CMR) increased from 2.95 per 100,000 in 1973–1975, to 3.53 per 100,000 in 1990–1992, and 5.67 per 100,000 in 2004–2005, and age-standardized morality rates (ASMR) increased from 2.98 per 100,000 in 1973–1975 to 3.08 per 100,000 in 1990–1992, and 3.85 per 100,000 in 2004–2005.

**Figure 2:**
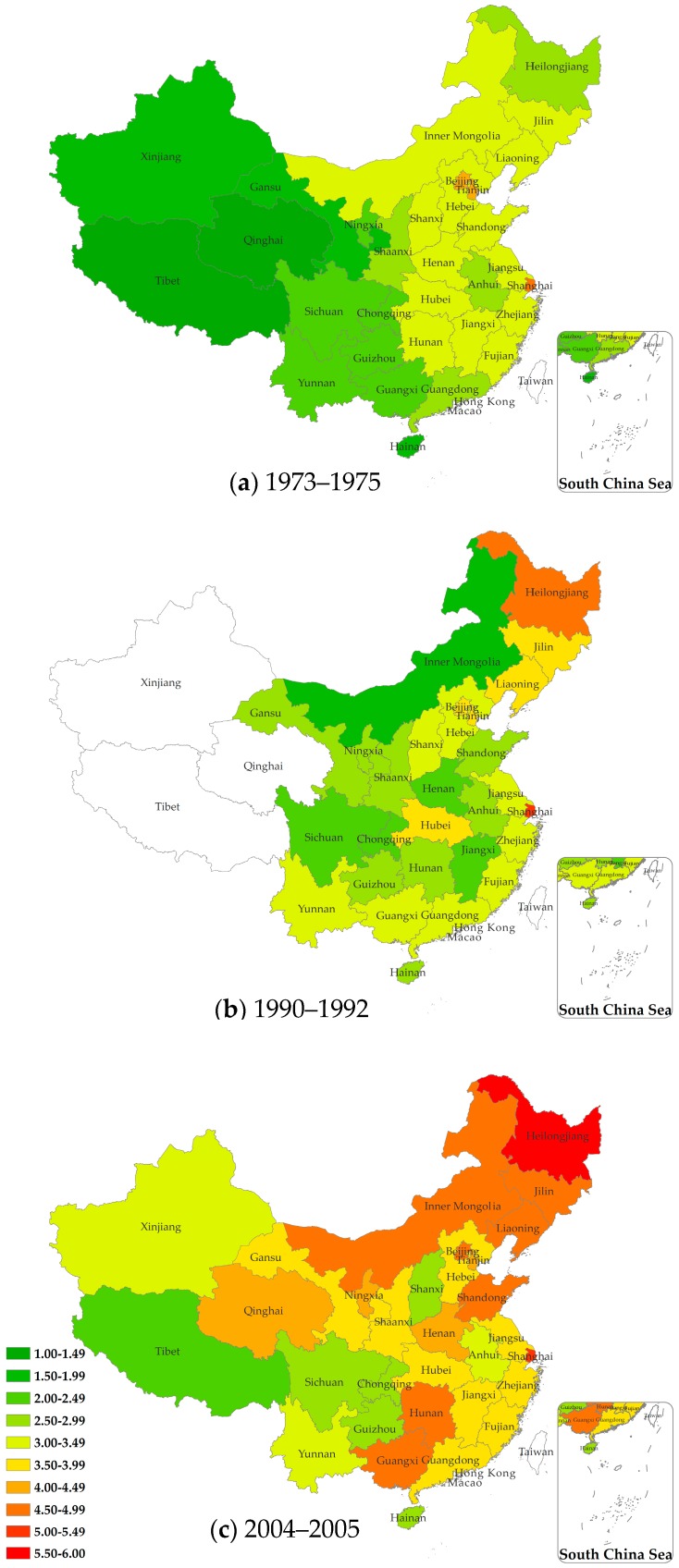
Female breast cancer age-standardized morality rates (ASMRs) by province (excluding Taiwan, Hong Kong, Macau) as recorded by three mortality surveys: (**a**) 1973–1975 the First National Survey of Death Causes; (**b**) 1990–1992 the Second National Retrospective Sampling Survey of Death Causes, with Xinjiang, Qinghai, Tibet not included; and (**c**) 2004–2005 the Third National Retrospective Sampling Survey of Death Causes. Map source: Sino Maps Press.

Breast cancer ASMRs by province for the three mortality surveys are shown in Figure 2. Values of these provincial ASMR estimates are presented in online Appendix C Table C1. In the period 1973–1975, Shanghai, Beijing and Tianjin had the highest ASMR (4.25 per 100,000 to 4.80 per 100,000), while the provinces in the Southwest and Northwest had lower rates (from Tibet: 1.23 per 100,000; Qinghai: 1.48 per 100,000 to 1.92 and 1.97 per 100,000 for Hainan and Xinjiang respectively). In the period 1990–1992, Shanghai still had the highest breast cancer ASMR (5.03 per 100,000), and the rates for three provinces in the Northeast as well as Beijing, Tianjin and Hubei were also higher than other provinces. In 2004–2005, the areas with the highest breast cancer mortality rates were Shanghai (5.21 per 100,000) and Heilongjiang Province (5.69 per 100,000), while the mortality rates recorded in Liaoning, Jilin, Shandong, Guangxi, and Hunan (ranging from 4.53 to 4.84 per 100,000) were higher than those seen in the neighboring provinces.

### 3.2. Geographical Variation over Time

**Figure 3:**
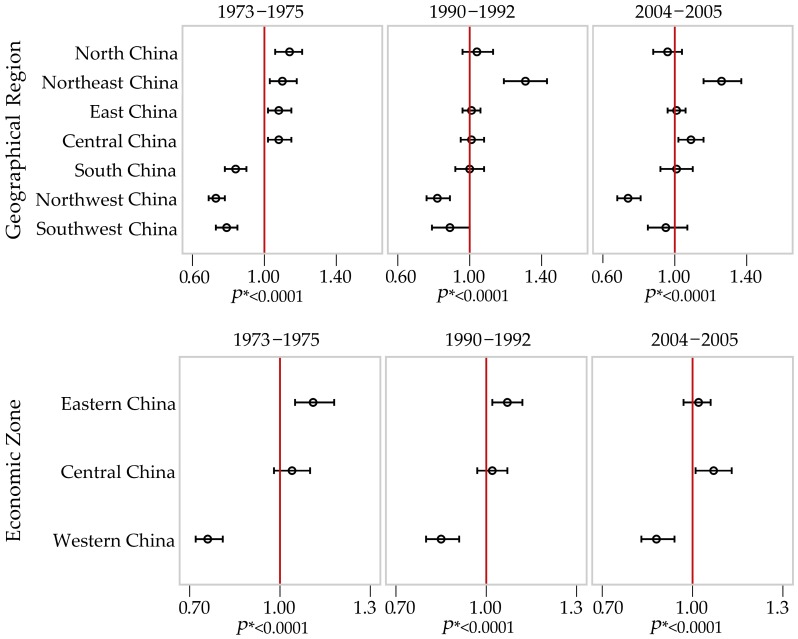
Rate ratios (RR) and 95% confidence intervals for female breast cancer mortality in China by geographical region and economic zone across three time periods, 1973–1975, 1990–1992 and 2004–2005, with the national average being the reference (1.00). * *p*-value for the effect of geographical unit.

Estimates of the rate ratios (RR) for female breast cancer death across seven geographical regions over the three surveys are shown in the upper panel in Figure 3. Values of these RR estimates and their 95% confidence intervals, by geographical regions and economic zones are presented in online Appendix D Table D1. Overall, the variation in breast cancer mortality was significant (*p* < 0.0001) between the seven geographical regions for each of the survey periods. Women living in the Northwest and Southwest areas had a lower risk of breast cancer mortality than the national average for all three survey periods, although there was an increase in risk over time, while women living in the Northeast had a higher rate than the national average for all three survey periods. The interaction between geographical region and death survey period was significant (*p* = 0.002), indicating that the geographical differential had widened over time.

When we looked at breast cancer mortality across the three economic zones (the lower panel in Figure 3), the results were consistent with those by the seven geographical regions. The Eastern economic zone had a higher breast cancer mortality rate than the national average, while the Western zone had a lower rate. The difference in the risk of breast cancer mortality in the three zones showed some gradual decrease over time, with the interaction between economic zone and survey period being non-significant (*p* = 0.089), but the geographical differential remained significant in each survey period (*p* < 0.001).

### 3.3. Correlation Analysis

**Figure 4:**
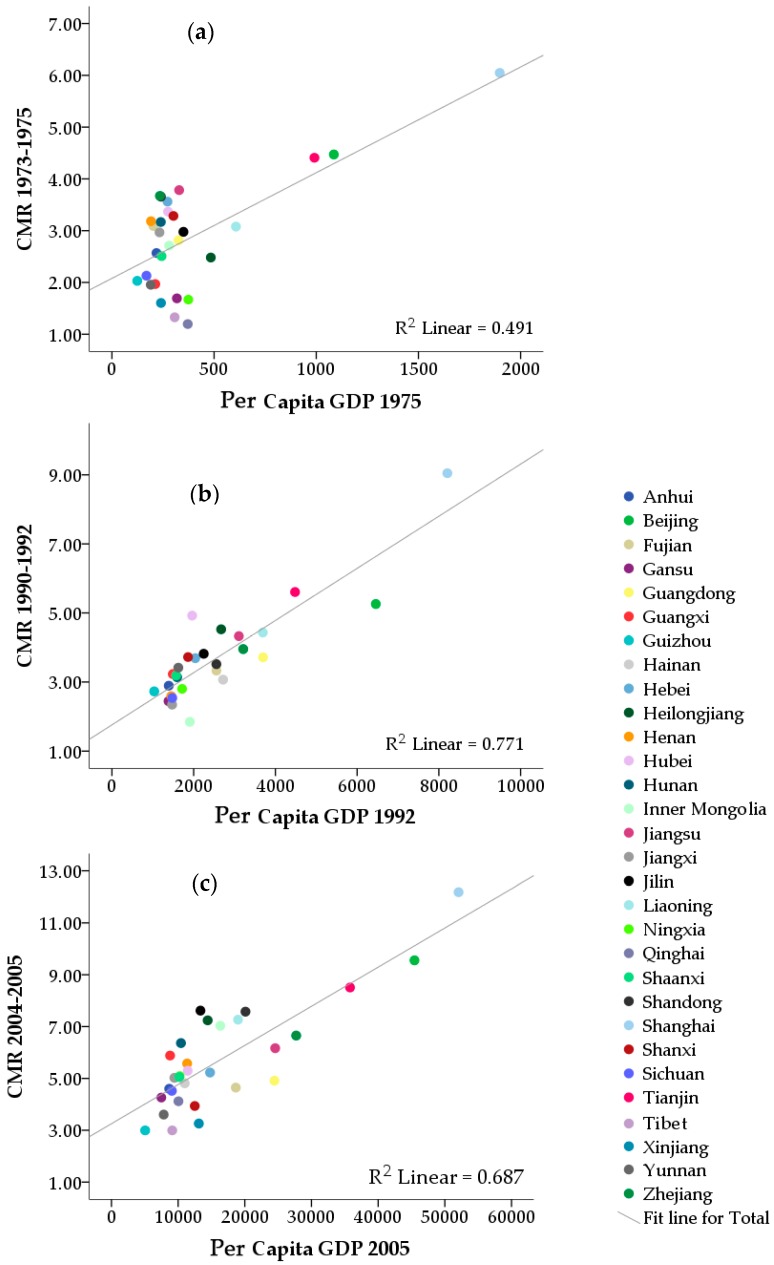
Scatterplot for the crude mortality rate (CMR) of breast cancer and per capita GDP by province. The linear relation between breast cancer CMR over the period: (**a**) 1973–1975 and per capita GDP for 1975 across 30 provinces; (**b**) 1990–1992 and per capita GDP for 1992 across 27 provinces; (**c**) 2004–2005 and per capita GDP for 2005 across 30 provinces.

Figure 4 shows scatterplots for the CMR of breast cancer and per capita GDP by province across each of the three death survey periods. The coefficient of determination (R^2^ value) is also presented. We found a moderate to high correlation between CMR of breast cancer and GDP over time.

The correlation between CMR of breast cancer and NHHCB per 10,000 people was significant at the 0.05 level for the two more recent survey periods 1990–1992 (r = 0.68) and 2004–2005 (r = 0.71), but was not significant for the period 1973–1975 (r = 0.28). The correlation between CMR of breast cancer and NPD per 10,000 people was significant across all three time periods, with the correlation coefficients being 0.40 for the period 1973–1975, 0.65 for 1990–1992, and 0.70 for 2004–2005.

Pearson correlation tests showed that there was a significant correlation between the breast cancer CMR and the average number of live births for women aged 15–64 for 1973–1975 (r = −0.82, *p* < 0.001), 1990–1992 (r = −0.73, *p* < 0.001) and 2004–2005 (r = −0.80, *p* < 0.001).

## 4. Discussion

As breast cancer is the most frequently diagnosed cancer in Chinese women, and a leading cause of cancer related death, there is a strong need for a greater understanding of the epidemiology of the disease in China. Whilst previous research has indicated that there are some distinct geographical patterns in the incidence and mortality of breast cancer in China [20], and that these patterns have changed over time [10], this study is, to our knowledge, the first to examine both temporal trends from the mid-1970s to the mid-2000s and the geographical variation in breast cancer mortality amongst Chinese women. The results of this study demonstrate that there was significant regional variation in each of the survey periods and that this regional differential had widened over time when we looked at breast cancer mortality across seven geographical regions in China.

Our results identified three important patterns. First, we found that across the country the overall mortality rate for female breast cancer increased over time (Figure 2). From the mid-1970s to early 1990s, the increase was only moderate (3.4%), but from the early 1990s to the mid-2000s, a relatively dramatic increase was observed (25.0%). An additional analysis indicates that this increasing temporal trend was significant (*p* < 0.001). These results are consistent with the latest data, which showed that the age-standardized female breast mortality rate in China increased by 1.1% per year from 2000 to 2011 [21]. This rise in the breast cancer mortality rate was observed across all provinces over the three study periods, and most studies also predict that this upward trend will continue in the foreseeable future [3,4,22].

Second, geographical variations in breast cancer mortality rates among Chinese women were observed for each period and increased over the three death survey periods from 1973–1975 to 2004–2005. Variation was found by province as shown in the maps of Figure 2, and by geographical region, and economic zone (Figure 3). Overall, breast cancer mortality rates were higher in large cities (Beijing, Tianjin, and Shanghai) and in the Northeast provinces than those observed in the Northwest and Southwest regions. The general trends we reported are similar to those found in previous studies [5,23], while our study also builds on the previous results by using cause of death survey data representative of the national population in each survey period and by including data from before the economic reforms that occurred in China in the late 1970s.

Lastly, we found that these temporal and spatial trends in breast cancer mortality were correlated with several social and economic measures. We found that the average per capita GDP level, and the level of medical facilities and number of practicing doctors in each province, had a positive correlation with the mortality rate of breast cancer in that province. It seems that high socioeconomic level is associated with high breast cancer mortality. This kind of association has previously been reported in China [24], but the opposite association has been found in many Western countries, with women of higher socioeconomic level having a lower mortality rate, mainly due to earlier diagnosis and more appropriate treatment, which then results in an increased breast cancer survival rate [25,26,27]. We also found that the average number of live births amongst women aged 15–64 in each province had a negative correlation with the mortality rate of breast cancer in each province, and that the average number of live births for Chinese women aged 15–64 decreased substantially over time (2.62 in 1982, 2.10 in 1990 and 1.35 in 2000, respectively). A previous meta-analysis of 14 studies showed that term birth is a protective factor for breast cancer in Chinese women, and that increasing numbers of term births will decrease the risk of breast cancer [28]. Women who were nulliparous were at increased risk of luminal breast cancer, whereas women with more than two children had a decreased risk [29]. We found that regions with a lower average number of live births, such as Beijing, Tianjin, Shanghai, had a higher CMR of breast cancer, while the regions with higher average number of live births such as Guizhou, Yunnan, Ningxia, had a lower CMR of breast cancer.

Temporal trends in breast cancer mortality can be explained by improved screening and treatment, as well as changes in exposure to risk factors. Because there is neither an organized nationwide screening program nor national breast cancer screening guidelines in China [30], changes in risk factors may contribute substantially to the temporal trends observed. In the same period in which we observed rising mortality trends for breast cancer, the GDP of China increased from 365 billion in 1978 to 18,458 billion in 2005, with an average annual economic growth rate of 11.56% [15]. The overall increase in breast cancer mortality from the mid-1970s to the mid-2000s in China is possibly a consequence of this increase in economic level in China, which then resulted in a shift in lifestyle (reduced levels of physical activity) and dietary habits (from a low-fat, high vegetable diet to a high-fat and animal protein diet) in the past few decades in China. It has been well documented internationally that higher body weight and lower levels of physical activity are associated with a greater risk of breast cancer incidence and mortality [31], and a Chinese study has also reported an association between body size, fat distribution and breast cancer incidence [32]. As a result of the increasing intake of Western style food and lack of exercise, the proportion of Chinese women who were overweight or obese increased over time from 4.6% in 1991 to 11.0% in 2011 [33]. In general, it is also likely that levels of physical activity amongst Chinese population decreased over time in the cities and more developed areas, while women in rural and less developed areas are still more likely to be undertaking more physical work than those living in more urbanized areas [34]. It is possible that these changes were another factor in the increasing number of breast cancer deaths we observed. It is also likely that the dramatic changes in reproductive history which have occurred in China (lower fertility rate and delayed first pregnancy), and especially the birth-control policies introduced in the mid-1970s, are a unique factor in this upward trend [3,35,36]. This possibility is supported by our finding that there was a negative correlation between the average number of live births and breast cancer mortality rate. The patterns in breast cancer mortality between geographical areas that we observed also reflect these changes.

As expected, we found that geographical variation in breast cancer mortality decreases as the geographical unit used increases (Figure 2 and Figure 3). The variation is largest when the analyses use province (*n* = 31), as a unit, is reduced substantially when the larger unit of geographical region (*n* = 7) is used, and is reduced even further when the whole nation is divided into three economic zones. However, the geographical variation observed was significant for all analyses no matter which geographical unit was used. The overall consistency of these results suggests these findings are reliable. Women in Western China had a lower risk of dying from breast cancer for the whole study period, while the risk ratio for women in Eastern China decreased significantly over time from higher in the first two survey periods to being closer to the national average in the most recent period. The opposite is true for Central China, with the risk increasing over time, so that, by the most recent period, it was significantly higher than the national average.

There are some possible explanations for the changes in breast cancer mortality across geographical regions that we observed. Geographical differences in cancer mortality may be the result of differences in access to cancer care, or differences in the rate of incidence of breast cancer in the area, or a combination of the two. The changing trends in breast cancer mortality over time for the three economic zones represent three typical patterns according to socioeconomic development levels. First, Eastern China includes large cities (Beijing, Shanghai and Tianjin) and coastal provinces, is an area with relatively high socioeconomic level and experienced the fastest economic growth from the economic reforms introduced in the late 1970s. As this area had a relatively high socioeconomic level even before the economic reform and the rapid growth after that, women living in Eastern China have historically had a higher risk of developing breast cancer, which resulted in higher mortality rates, as observed in the periods 1973–1975 and 1990–1992. Conversely, women in Eastern China generally have higher survival rates after diagnosis due to the fact that this area has the highest level of medical facilities in China [37], and women from advantaged areas tend to present with earlier stage disease than women from more disadvantaged areas [38]. This offsets the higher incidence rates, and leads to a reduced mortality rate close to the national average in the most recent survey period. Second, Western China includes inland provinces with historically a much lower economic development level, and which benefitted much less from the economic reforms, so that per capita GDP in Western China is less than half of that of Eastern China. As a result of this lower level of socioeconomic development, the risk of developing breast cancer was much lower among women in the west, which was likely the main reason for the lower mortality rates observed in this study for all three death survey periods. However, over time, the mortality rates in this economic zone were rising faster than in Eastern China, probably due to the generally lower level of medical facilities and lack of accessibility to top level medical care. Third, breast cancer mortality rates in Central China were generally somewhere in-between the other two economic zones, so that the mortality rates in the mid-1970s and early 1990s were similar as the national average. However, in the most recent period (2004–2005), the mortality rates became significantly higher than the national average. The constant increase in the incidence rate and the generally low survival rate with breast cancer (compared with women in more developed areas) are both contributing to the continued increase in the breast cancer mortality rate for women in Central China. Thus, while improved survival has led to a reduction in the pace of the increase in the mortality rate in areas of higher socioeconomic status such as Eastern China, the impact of the changes brought about by China’s economic development on breast cancer mortality rates is more obvious in the Western region of China, where breast cancer mortality rates have been increasing more quickly.

Our study had some possible limitations. First, although the sample sites covered by the two sampling surveys, which collected information on deaths occurred in the study years, are representative of the country as a whole [39], the population covered is relatively small and only a limited number of sites were selected from each province in the second and third national retrospective sampling death surveys [10]. Thus, the provincial mortality rates reported here may be an overestimate or underestimate for that province. Second, there were three provinces for which data was not available in the Second Survey (1990–1992), Xinjiang, Qinghai and Tibet, since most sampling counties in those provinces could not complete the survey and data were deemed invalid for analysis [12]. This may mean there is some bias in the rate for the Western area of China in that period. Furthermore, we grouped data based on where women live, thus it is possible that inferences at the geographical regional level do not transfer directly to individuals. Finally, as we acknowledged in a recent publication [21], in a country of 1.4 billion people, with a sizeable population of migrant workers (9% of the population), there are many challenges to ensuring that the mortality numerator data represents the same population at risk as the estimated resident population denominator, particularly when considering cases treated in major urban facilities and migrant workers from rural areas. The data used in this study was based on the place of permanent residence, not place of treatment. Despite these limitations, the data from the three national surveys on cause of death provides valuable information that we believe is currently underutilized [40].

## 5. Conclusions

We found that overall breast cancer mortality increased significantly from the mid-1970s to the mid-2000s in China. We also found that there were significant geographical variations in breast cancer mortality over the same time period and that the geographical differential had widened over time. Our findings of large differences in breast cancer mortality between geographical areas, changes in geographical patterns over time, and the correlation we observed between various economic measures and breast cancer mortality do suggest that there are differences in cancer prevention, diagnosis and care across China. If this is indeed the case, it may be possible to reverse these trends and reduce the widening geographical inequalities in breast cancer mortality with both prevention strategies, such as public health campaigns to promote healthy lifestyle choices [4] and more equitable access to diagnostic and treatment facilities.

## Appendix A

**Seven geographical region** (Figure 1a)

Provinces in mainland China were grouped into seven geographical regions based on geographical location in this analysis:

1. **North China** (Beijing, Tianjin, Shanxi, Hebei, Inner Mongolia Autonomous Region)
2. **Northeast China** (Heilongjiang, Jilin, Liaoning)
3. **East China** (Shanghai, Jiangsu, Zhejiang, Anhui, Jiangxi, Shandong, Fujian)
4. **Central China** (Henan, Hubei, Hunan)
5. **South China** (Guangdong, Guangxi, Hainan)
6. **Southwest China** (Chongqing, Sichuan, Guizhou, Yunnan, Tibet)
7. **Northwest China** (Shaanxi, Gansu, Qinghai, Ningxia Hui Autonomous Region, Xinjiang Uygur Autonomous Region)

**Three economic zone** (Figure 1b)

According to the level and the speed of development in economic, China also been divided into three major economic zones:

The Eastern China zone includes Tianjin, Beijing, Hebei, Liaoning, Shanghai, Jiangsu, Zhejiang, Fujian, Shandong, Guangdong, and Hainan.

The Central China zone includes Shanxi, Jilin, Heilongjiang, Anhui, Jiangxi, Henan, Hubei, and Hunan.

The Western China zone includes Chongqing, Sichuan, Guizhou, Yunnan, Guangxi, Tibet, Shaanxi, Gansu, Ningxia, Inner Mongolia, Qinghai, and Xinjiang.

## Appendix B

**Table B1:** Characteristics of the study population.

	Number of Deaths	Population	Per Capita GPD	Number of Deaths	Population	Per Capita GPD	Number of Deaths	Population	Per Capita GPD
	1973–1975			1990–1992			2004–2005		
**Province**									
Beijing	533	11,921,385	1086	80	1,521,398	6458	120	1,255,754	45,444
Tianjin	441	10,003,046	991	68	1,213,045	4481	113	1,328,365	35,783
Hebei	2493	70,018,076	272	349	9,461,858	2040	209	3,998,627	14,782
Shanxi	1074	32,697,776	301	120	3,224,205	1862	124	3,149,896	12,495
Inner Mongolia	306	11,286,804	280	22	1,189,805	1906	94	1,336,708	16,331
Liaoning	1605	52,136,241	607	183	4,130,045	3693	285	3,921,907	18,983
Jilin	1019	34,233,741	350	142	3,720,631	2246	155	2,035,309	13,348
Heilongjiang	1010	40,709,250	484	182	4,022,425	2672	190	2,623,978	14,434
Shanghai	985	16,287,701	1898	183	2,022,405	8208	103	845,386	52,060
Jiangsu	3126	82,630,955	329	492	11,367,746	3106	537	8,708,157	24,560
Zhejiang	1894	51,588,553	235	350	8,855,355	3212	431	6,478,093	27,703
Anhui	1646	64,089,979	218	231	7,985,474	1390	252	5,477,507	8670
Fujian	949	30,708,605	203	150	4,505,502	2557	154	3,314,821	18,646
Jiangxi	1222	41,191,696	233	124	5,290,316	1472	164	3,265,107	9440
Shandong	1963	53,688,310	240	363	10,323,297	2556	507	6,692,702	20,096
Henan	3134	98,471,373	191	284	10,955,314	1452	490	8,789,059	11,346
Hubei	2045	60,725,263	274	504	10,237,560	1962	291	5,495,822	11,431
Hunan	2213	69,870,339	239	296	9,424,817	1595	267	4,196,179	10,426
Guangdong	1777	63,123,805	326	417	11,227,881	3699	257	5,230,642	24,438
Guangxi	706	35,877,301	212	208	6,441,808	1490	245	4,164,893	8788
Hainan	127	5,711,874	326	26	847,327	2719	35	727,437	10,998
Sichuan	2811	132,015,851	169	322	12,685,421	1477	213	5,073,707	9060
Guizhou	723	35,608,036	124	148	5,424,985	1034	53	1,769,571	5052
Yunnan	813	41,588,565	190	197	5,768,515	1625	140	3,884,535	7835
Tibet	17	1,281,431	307	*	*	1468	6	200,357	9114
Shaanxi	892	35,584,375	243	148	4,642,551	1571	100	1,979,434	10,161
Gansu	369	21,802,163	318	*	*	1384	101	2,370,347	7477
Qinghai	57	4,765,301	371	*	*	1912	17	412,737	10,045
Ningxia	80	4,795,636	374	127	5,190,772	1718	36	707,641	10,239
Xinjiang	241	15,028,025	240	31	1,106,540	2477	48	1,472,894	13,108
**Geographical Region**									
North China	4847	135,927,087		639	16,610,311		660	11,069,350	
Northeast China	3634	127,079,232		507	11,873,101		630	8,581,194	
East China	11,785	340,185,799		1893	50,350,065		2148	34,781,773	
Central China	7392	229,066,975		1084	30,617,691		1048	18,481,060	
South China	2610	104,712,980		651	18,517,016		537	10,122,972	
Southwest China	4364	210,493,883		667	23,878,939		558	13,799,681	
Northwest China	1639	81,975,500		306	10,939,863		302	6,943,053	
**Economic zone**									
Eastern China	15,893	447,818,551		2661	65,475,829		2875	45,651,787	
Central China	13,363	441,989,417		1883	54,860,742		1809	31,882,961	
Western China	7015	339,633,488		1203	42,450,415		1199	26,244,335	

* data not available.

## Appendix C

**Table C1:** Age-standardized mortality rate of breast cancer by province as recorded by three mortality surveys in China: 1973–1975, 1990–1992 and 2004–2005.

Code	Name	Areas	1975	1992	2005
110000	北京市	Beijing	4.32	3.71	4.73
120000	天津市	Tianjin	4.25	3.76	4.36
130000	河北省	Hebei	3.20	3.28	3.55
140000	山西省	Shanxi	3.17	3.43	2.58
150000	内蒙古自治区	Inner Mongolia	3.34	1.74	4.80
210000	辽宁省	Liaoning	3.40	3.72	4.59
220000	吉林省	Jilin	3.49	3.76	4.82
230000	黑龙江省	Heilongjiang	2.85	4.63	5.69
310000	上海市	Shanghai	4.80	5.03	5.21
320000	江苏省	Jiangsu	3.50	3.22	3.63
330000	浙江省	Zhejiang	3.40	3.02	3.84
340000	安徽省	Anhui	2.56	2.59	3.38
350000	福建省	Fujian	3.26	3.10	3.61
360000	江西省	Jiangxi	3.07	2.45	3.62
370000	山东省	Shandong	3.19	2.95	4.56
410000	河南省	Henan	3.09	2.37	4.16
420000	湖北省	Hubei	3.42	3.94	3.90
430000	湖南省	Hunan	3.27	2.91	4.55
440000	广东省	Guangdong	2.66	3.17	3.67
450000	广西壮族自治区	Guangxi	2.16	3.07	4.53
460000	海南省	Hainan	1.92	2.96	2.79
500000	重庆市	Chongqing	2.24	2.20	2.92
510000	四川省	Sichuan	2.24	2.20	2.92
520000	贵州省	Guizhou	2.16	2.80	2.52
530000	云南省	Yunnan	2.01	3.10	3.01
540000	西藏自治区	Tibet	1.23		2.45
610000	陕西省	Shaanxi	2.73	2.99	3.85
620000	甘肃省	Gansu	1.88	2.59	3.82
630000	青海省	Qinghai	1.48		4.15
640000	宁夏回族自治区	Ningxia	2.41	2.61	4.36
650000	新疆维吾尔自治区	Xinjiang	1.97		3.12

## Appendix D

**Table D1:** Estimates of mortality rate ratio (RR) * and 95% confidence interval (CI) of breast cancer among Chinese women by geographical area across three time periods, 1973–1975, 1990–1992, and 2004–2005.

	RR and 95% CI		
	1973–1975	1990–1992	2004–2005
**Geographical region**			
Northern China	1.14 (1.06–1.21)	1.04 (0.96–1.13)	0.96 (0.88–1.04)
Northeast China	1.10 (1.03–1.18)	1.31 (1.19–1.43)	1.26 (1.16–1.37)
East China	1.08 (1.02–1.15)	1.01 (0.96–1.06)	1.01 (0.96–1.06)
Central China	1.08 (1.02–1.15)	1.01 (0.95–1.08)	1.09 (1.02–1.16)
South China	0.84 (0.78–0.90)	1.00 (0.92–1.08)	1.01 (0.92–1.10)
Northwest China	0.73 (0.69–0.78)	0.82 (0.76–0.89)	0.74 (0.68–0.81)
Southwest China	0.79 (0.73–0.85)	0.89 (0.79–1.00)	0.95 (0.85–1.07)
*p*-value for the effect of geographical region	<0.0001	<0.0001	<0.0001
**Economic zone**			
East China	1.11 (1.05–1.18)	1.07 (1.02–1.12)	1.02 (0.97–1.06)
Central China	1.04 (0.98–1.10)	1.02 (0.97–1.07)	1.07 (1.01–1.13)
Western China	0.76 (0.72–0.81)	0.85 (0.80–0.91)	0.88 (0.83–0.94)
*p*-value for the effect of economic zone	<0.0001	<0.0001	<0.0001

* National average is set as the reference (1.00).
